# Long-Term Survival After Resection of Ductal Carcinoma in the Body and Tail of Pancreas

**DOI:** 10.1155/1990/78049

**Published:** 1990

**Authors:** Per Billesbolle, Lise G. Larsen, Flemming Burcharth, Helge Baden

**Affiliations:** 1 Department of Surgical Gastroenterology Herlev Hospital University of Copenhagen Denmark; 2 Department of Pathology Herlev Hospital University of Copenhagen Denmark; 3 Dept. D Herlev Hospital Herlev DK-2730 Denmark

## Abstract

We report on a 62-year-old male, who had resection of a large ductal carcinoma in the body and tail of the pancreas. Four months later a metastasis was removed from the abdominal scar, and 14 months later another metastasis was removed from the anterior wall of the stomach. Moreover, he had a left mastectomy followed by radiation therapy for a primary ductal carcinoma and a transurethral resection of the prostate because of benign hyperplasia. A minor focus of primary highly differentiated adenocarcinoma was found in the chips. More than 6 years later, the patient is without any signs of recurrences.


*HPB Surgery*, 1990, Vol. 2, pp. 51–55
Reprints available directly from the publisher
Photocopying permitted by license only

© 1990 Harwood Academic Publishers GmbH
Printed in the United Kingdom

### LONG-TERM SURVIVAL AFTER RESECTION OF DUCTAL CARCINOMA IN THE BODY AND TAIL OF PANCREAS

PER BILLESBOLLE[1], LISE G. LARSEN[2], FLEMMING BURCHARTH[1] and HELGE BADEN[1]

*Department of Surgical Gastroenterology[1] and Department of Pathology[2], Herlev Hospital, University of Copenhagen, Denmark.*

*(Received 17 January 1989; in final form 26 April 1989)*

We report on a 62-year-old male, who had resection of a large ductal carcinoma in the body and tail of the pancreas. Four months later a metastasis was removed from the abdominal scar, and 14 months later another metastasis was removed from the anterior wall of the stomach. Moreover, he had a left mastectomy followed by radiation therapy for a primary ductal carcinoma and a transurethral resection of the prostate because of benign hyperplasia. A minor focus of primary highly differentiated adenocarcinoma was found in the chips. More than 6 years later, the patient is without any signs of recurrences.

KEY WORDS: Pancreatic cancer, pancreatic resection

### INTRODUCTION

Five-year survival after resection for ductal carcinoma in the body or tail of the pancreas is extremely rare, and only four cases have been reported in the literature.

We report on a patient with proven ductal carcinoma of the pancreas, who is alive more than six years after pancreatic resection.

### CASE HISTORY

A 62-year-old male was admitted after half a year of intermittent pain in the upper left abdominal quadrant, malaise and rumbling of the bowels, but no weight loss. Physical examination revealed no abdominal mass. Chest X-ray was normal. Liver chemistry, hemoglobin, blood cell count, fatty acids, cholesterol, electrolytes and blood glucose were within normal limits. The sedimentation rate was slightly raised, and serum carcinoembryonic antigen was 16 micromol/1 (normal $<2.5$ micromol/1).

Ultrasonography demonstrated a solid mass in the tail of the pancreas and fine-needle biopsy showed malignant cells. Abdominal angiography showed a peripheral occlusion of the splenic artery.

At laparotomy a tumor measuring 11 by 10 by 7 cm was found in the body and tail of the pancreas. It compressed the splenic vessels and the middle colic artery. There

Correspondence to: Dr. F. Burcharth, Dept. D, Herlev Hospital, DK-2730 Herlev, Denmark.

were no liver metastases. Resection of the pancreatic body and tail, the transverse colon and splenectomy was performed. Regional lymph nodes removed during the operation, and one in the resected specimen were without metastases. In the resected lesser omentum and retroperitoneal tissue two small areas contained carcinoma. The postoperative course was uneventful.

Four months later a solitary metastasis was excised from the abdominal scar. Ten months later the patient had upper abdominal pain, and ultrasonography showed a 6 cm tumor in the anterior wall of the stomach. At laparotomy the tumor was adherent to the left colonic flexure, and resection of the stomach and colon were performed. Three lymph nodes removed during operation and four in the specimen were without metastases.

At twenty-one months a tumor occurred in the left breast, suspected to be a metastasis from the pancreatic cancer, and a mastectomy was performed. At thirty-one months left axillary lymph node dissection was performed because of metastases, and histological revision of the specimen showed ductal breast carcinoma. One of three lymph nodes contained carcinoma and radiation therapy was given. At thirty-nine months, a transurethral resection of the prostate was performed for hyperplasia.

All histological slides were reviewed. The primary tumor in the pancreas was a poorly differentiated ductal adenocarcinoma (Figure 1). There was no tumor invasion of veins or nerves and the resection lines were without cancer. The histology was also typical for a primary pancreatic adenocarcinoma, that other primary pancreatic tumors including endocrine and germinative tumors could be ruled out. The tumors in the abdominal scar and stomach were compatible with metastases from the pancreatic carcinoma. The breast tumor was a primary, poorly differentiated ductal breast carcinoma, and in the chips from the prostate a minor focus of primary, highly differentiated prostatic adeno-carcinoma was identified. Examination for estrogen receptors was not performed and cannot be done now on the formalin treated specimens.

After more than 6 years the patient is without any signs of recurrences, and maintains his weight in spite of exocrine pancreatic insufficiency with mild diarrhoea.

### DISCUSSION

Reviewing the literature, we found four putative long-term survivors after resection for ductal carcinomas in the body or tail of the pancreas[1–4].

The first recorded long-time survival after resection of a ductal carcinoma in the body of the pancreas was published in a Russian journal in 1922 and abstracted in English in 1923[1]. A 30-year-old woman had had upper abdominal pain for many years. Physical examination revealed a hard, mobile tumor in the left hypochondrium At laparotomy in 1913, a hard, nodular, mobile tumor, the size of two fists was localized behind the stomach. It was removed by a subtotal pancreatectomy leaving a 1 by 3 cm piece of the head of the pancreas. Microscopy showed a "cylindrical-cell carcinoma". The patient developed a mild pancreatic insufficiency but no diabetes. She was doing well up to 1918 when a hard tumor at the size of a pea was discovered in the left clavicle. Radiation therapy was given. The patient was doing fairly well up to 1922, when she was lost for follow-up.

The second case was operated upon in 1927[2]. The patient was a slim 54-year-old male who had had fatigue for two months and had noticed a lump in the upper left abdomen.

Physical examination revealed a prominent visible tumor in the left hypochondrium. At laparotomy a tumor the size of one and a half fist was found in the lesser omental sac. A subtotal pancreatectomy was performed, leaving behind a minute fragment of the pancreas in the duodenal concavity. It was necessary to resect the splenic artery and a part of the splenic vein as well as a part of the portal vein. Microscopy showed "columnar cell carcinoma". The patient did not develop diabetes, and almost 7 years after the operation he was in an excellent state of health.

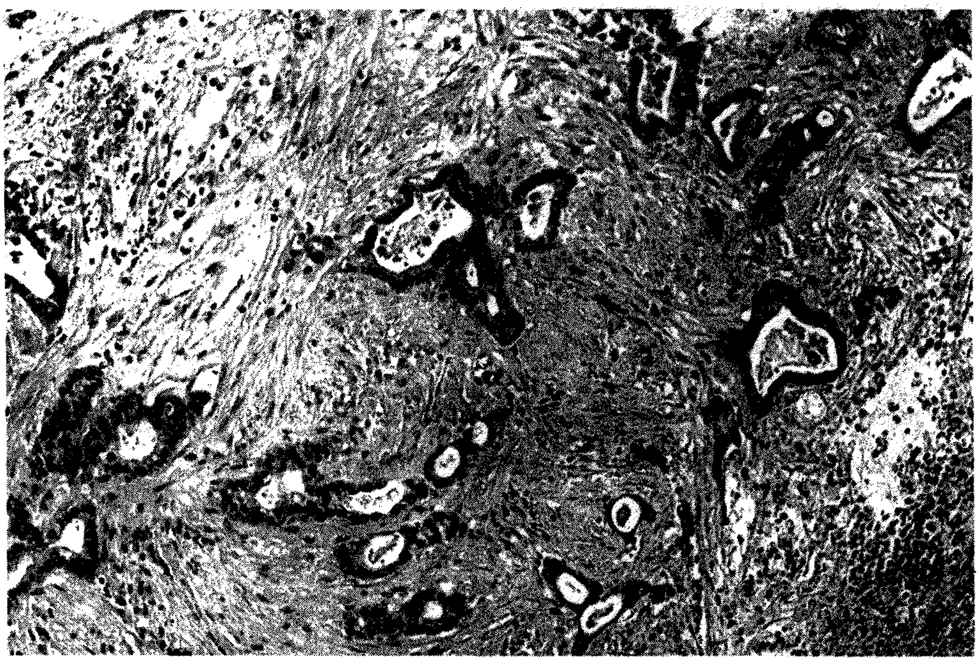

**Figure 1** Histologic appearance of the pancreatic cancer showing an infiltrating ductal carcinoma (H&E × 120).

The third case was from a series of cancer of the body and tail of the pancreas reported in 1971[3]. A woman was operated upon for a suspected pancreatic pseudocyst, but a small tumor was found in the distal pancreas. It was removed by a simple wedge resection. Microscopy showed adenocarcinoma. She was doing well 15 years later.

The fourth case appears from a table in a paper summarizing the results of surgical treatment of pancreatic cancer in 57 institutions in Japan[4]. The patient is the one who survived more than 5 years, but less than 10 years, among 66 patients who survived resections of cancer in the body or tail of the pancreas.

It is beyond any doubt that these four reported cases had cancer of the body or tail of the pancreas. However, because of the huge size of the two first cases they may have been cystadenocarcinomas which have a better prognosis than true ductal cancer. Further, endocrine and germinative tumors in the pancreas have good prognosis. Accordingly an exact histopathological diagnosis is mandatory.

Our patient, the 5th reported 5-year-survivor in three quarters of a century, showed two distinctive features. There was two instances of late metastasis (abdominal wall and stomach) amenable to surgery and primary cancers in the prostate and breast supporting the theory, that pancreatic cancer may be a sex-hormone dependent tumor[5]. The hormone dependency was not known to us at the time of surgery, and delayed hormone receptor analysis was not possible.

Of patients with ductal carcinomas of the pancreatic body and tail, only a few seem to benefit from pancreatic resection, as most patients die within one year.

Only by detailed reporting on long-term survivors, it will be possible to decide if they possess unique characteristics.

*Acknowledgement*

Supported by a grant from the Boel-Foundation

*References*

1. Grekow, I.I. (1923) Surgery of the pancreas. The diagnosis and treatment of primary carcinoma of the pancreas, particularly of the body and tail of the gland. *Surg Gynecol Obstet*, **36**, 327.
2. Gordon-Taylor, G. (1934) The radical surgery of cancer of the pancreas. *Ann. Surg.* **100**, 206–214.
3. Goyanes, A.D. and Pack, G.T. (1971) Cancer of the body and tail of the pancreas. *Rev. Surg.* **28**, 153–175.
4. Nakase, A., Matsumoto, Y., Uchida, K. and Honjo, I. (1977) Surgical treatment of cancer of the pancreas and the peri-ampullary region: Cumulative results in 57 institutions in Japan. *Ann. Surg.* **185**, 52–57.
5. Greenway. B.A. (1987) Carcinoma of the exocrine pancreas: a sex hormone responsive tumour? *Br. J. Surg.* **74**, 441–442.

### INVITED COMMENTARY

The authors have made a contribution in demonstrating the incredible infrequency of cure of this specific lesion. Surgeons, however, must not abandon attempts for cure. Whereas this commentator has not achieved cure of a ductal cancer of the tail of the pancreas, he has achieved a four year survivor plus longer term survivors of patients with carcinoid and patients with mucinous cystadenocarcinoma of the tail. Histological subclassification of pancreatic (exocrine) cancers may give further prognostic information. Anatomic localization of some of the cancers which present on the cephalic aspect of the pancreas, along the portal vein, may be indefinite as to whether they originate in the "head or body" but it is obvious that the prognosis is bad.

Although the outlook for immediate progress is not bright, the combination of ERCP and serum Ca19-9 determination may provide additional survivors. Perhaps determination of Ca19-9 titer in pancreatic juice, collected via E.R.P. cannulation, might give helpful information.

The tumor and the patient were unusual as reflected in the long term survival and the multiplicity of tumors. In the commentator's experience, the occasional long term survivor of pancreatic cancer is usually female in spite of the fact that pancreatic cancer is more frequent in the male. The patient being reported had carcinomas of the breast and prostate, each obviously sex related in occurrence and in response to hormone therapy. Although estrogen has been shown to be involved in pancreatic metabolism, sex hormones have not been clearly implicated in either the causation or therapy of pancreatic ductal carcinoma. We routinely assay pancreatic cancers for estrogen and progesterone receptors but the findings are usually negative. Numerous studies, especially in Europe, are underway to explore the hormonal parameters of pancreatic cancer. Although potentially quite important, these studies to date do not yet provide effective clinical application.

John M. Howard
Professor of Surgery
Medical College of Ohio, USA

### INVITED COMMENTARY

This is indeed a remarkable case. The relatively long history (half a year), the pre-operative angiographic evidence of splanchnic infiltration, the considerable size of the tumor (11 × 10 × 7cm) and its infiltration of the retro-peritoneum – all of these findings really weighed the odds against resectability, let alone long-term survival.

And yet this patient did survive not only an extensive resection of the primary tumor (including transverse colon and spleen), but also two further resections of metastases 4 and 10 months later.

And then this male patient went on to develop mammary and prostatic cancers, both of which were also cured by surgery.

Returning to this adenocarcinoma of the body and tail of the pancreas, the authors have rightly stressed the importance of verification of the histology. As we all know: "Even if you admire your pathologist as much as I do mine you cannot give him your full trust on pancreatic histology" (Harbrecht 1974). If this is true – and I think it is – I would suggest, that the histological slides of this case be sent to an independent pathologist for a second opinion. And then it might be possible to examine the preparations by immunohistochemical studies, just to rule out the possibility of some form of malignant apudoma.

The authors' allusion to sex-hormone dependence of pancreatic tumors is pertinent to this case, but it lacks practical significance for this patient. So far none of the studies using tamoxifen have shown any significant effect on long-time survival (Greenway 1988). It is remarkable (and commendable) that the authors apparently resisted the temptation of treating this patient with adjuvant chemotherapy. It is worth commenting that this remarkable patient was cured of carcinoma of the pancreas by surgery alone.

M. Trede
Professor of Surgery
Kllinikum Mannheim
University of Heidelberg

